# A Bayesian Account of the Phantom Lighthouse Illusion

**DOI:** 10.1162/OPMI.a.390

**Published:** 2026-10-03

**Authors:** Charles Kemp, Weilun Xu, Simon J. Cropper

**Affiliations:** Melbourne School of Psychological Sciences, University of Melbourne, Melbourne, Australia; School of Computer and Communication Sciences, École Polytechnique Fédérale de Lausanne, Lausanne, Switzerland

**Keywords:** vision, depth perception, illusions, Bayesian inference, virtual reality

## Abstract

Observers sometimes infer that a rotating lighthouse beam originates from a “phantom lighthouse” that lies in the opposite direction from the true source of the beam. We argue that this illusion arises from underestimating the distance of the remote parts of the beam. This underestimation is consistent with Bayesian inference based on natural scene statistics, and also explains why lighthouse beams are perceived as pointing down rather than parallel to the ground. Our theory makes the novel prediction that the phantom lighthouse illusion emerges at a critical point at which the observer is located around 67 metres in front of the true lighthouse, and we find support for this prediction in a virtual reality experiment. Although we focus on the phantom lighthouse illusion, our approach contributes to a broader body of work that explains perceptual phenomena (including visual illusions) in terms of Bayesian inference.

## INTRODUCTION

Our visual systems mostly provide us with reliable information about the world, but occasionally they do not. Visual illusions are clearly important for understanding where and why our perceptual systems make errors, but are also valuable because they expose the mechanisms and principles that mostly support accurate perceptual inferences (Gregory, 2006). Here we add to a body of work which argues that visual illusions are expected when a near-optimal perceptual system is applied to stimuli that depart from the statistical structure of natural scenes (Geisler & Kersten, 2002; Khoei et al., 2017; Weiss et al., 2002; Yildiz et al., 2022; Zhu & Ma, 2017). We do so by presenting a simple Bayesian model and showing that it accounts for an illusion involving the perception of lighthouse beams in both natural and virtual environments.

Under some circumstances, an observer standing with her back to a lighthouse will perceive the beams sweeping over her head as originating from a phantom lighthouse somewhere far away in front of her (Figure 1a; see also Supplementary Movie S3). Although this phantom lighthouse illusion has received relatively little attention, we find it striking for two reasons. First, the illusion is readily experienced in everyday settings without specialized equipment. On Lady Elliot Island (Australia), staff report that visitors routinely ask about illusory lighthouses that appear to be visible off the shore of the island, and Somerville (2020, p. 61) confirms that the rotating beams of the Lady Elliot lighthouse are “commonly mistaken for other lighthouses over the horizon.” Second, the illusion is notable for the sheer magnitude of the inferential error made by the visual system. In the experience of the first author, the distance between the locations of the true and phantom lighthouses can amount to tens of kilometres.

**Figure 1.**
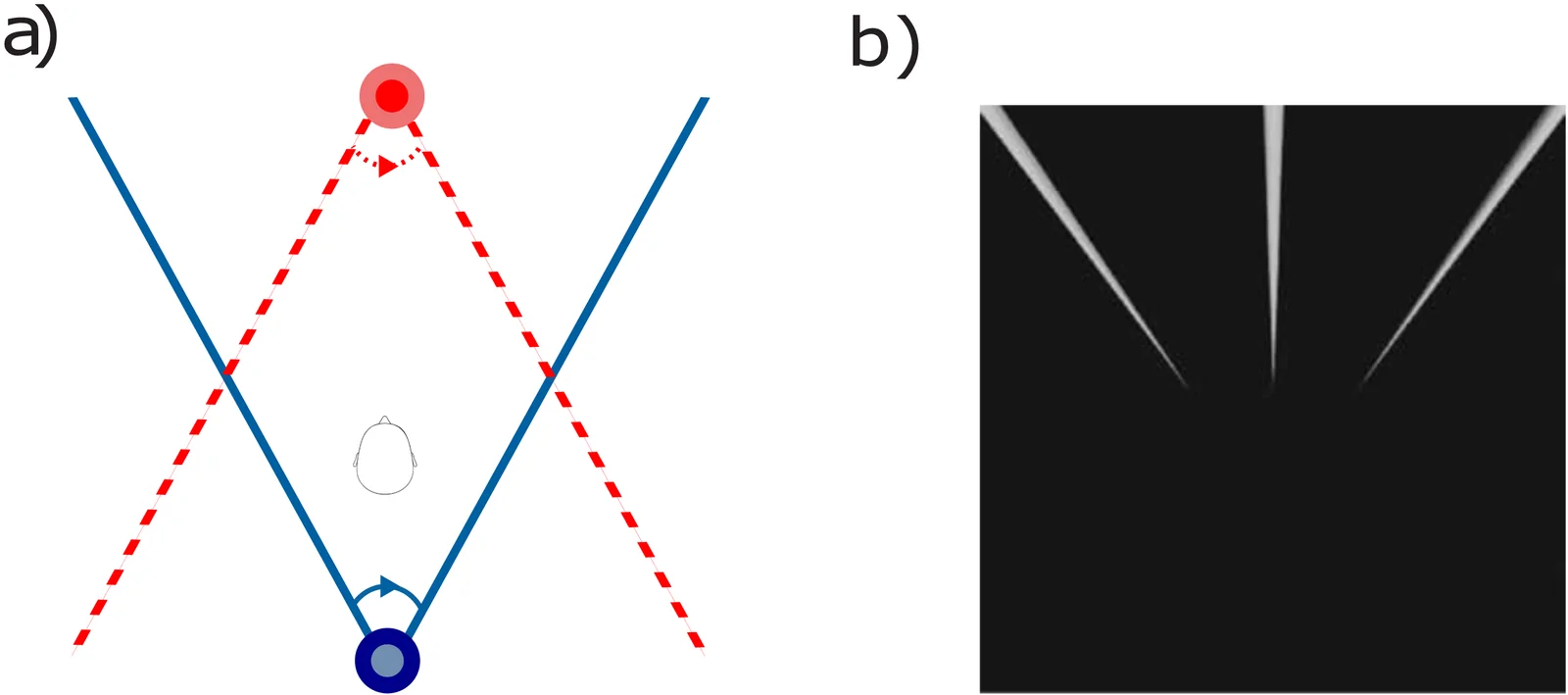
The phantom lighthouse illusion. (a) Bird’s eye view (not to scale) of an observer standing with her back to a lighthouse (blue circle). The observer perceives the beams sweeping clockwise over her head (blue lines) as originating from a “phantom lighthouse” (red circle) somewhere out in front of her. (b) Composite view showing three snapshots of the rotating beam as experienced by the observer in panel (a). In reality, the most distant points along the beams are far from each other, but the observer infers that the beams converge and meet at a distant source. The projection assumes that the separation between observer and lighthouse is 67 m, and the snapshots are separated by angles of 15°.

The earliest discussions of the phantom lighthouse illusion that we know date from the 1930s (Colange & Le Grand, 1937, 1938; Dunoyer, 1937; Rosenthal, 1938), and this illusion is also discussed by Minnaert (1993, p. 171), who describes it as a “most impressive sight”, and by Floor (1982, p. 233), who refers to it similarly as the “most impressive optical illusion associated with lighthouses”. Across this literature, three factors are proposed as contributing to the illusion. The first and most commonly-mentioned factor is an inadequate allowance for visual perspective (Colange & Le Grand, 1937; Floor, 1982; Minnaert, 1993; Rosenthal, 1938). In Figure 1b, for example, three lighthouse beams appear to converge in the image plane as a result of perspective projection, and an observer who takes convergence in the image as evidence for real-world convergence may incorrectly conclude that the beams are emitted from a source lying at the apparent point of convergence. Minnaert (1993) briefly mentions the “underestimation of faraway distances” as a second relevant factor, and suggests that the illusion depends in part on the perception that the beams do not extend infinitely but rather approach a point some finite distance away. A third factor is introduced by Colange and Le Grand (1937), who suggest that the photometric brightness of the beams is largest towards the horizon, and that the source of the beams is inferred to lie at the region of greatest brightness.

Here we focus on the second factor—“the underestimation of faraway distances”—and argue that this underestimation occurs because the observer relies on Bayesian inference using a prior derived from natural scene statistics. Minnaert (1993) devotes only a single sentence to this factor, and we go beyond his anecdotal description by implementing a formal model and showing that it accounts for the phantom lighthouse illusion. We also derive and test a key prediction that distinguishes our model from a *naive projection* account that attempts to explain the phantom lighthouse illusion based on convergence of the beams in the image plane. The projection in Figure 1b shows the view of an observer located 67 m in front of a lighthouse, and the three projected beams only become parallel in the image plane when the separation between observer and lighthouse is reduced to 0 m.^1^ The naive projection account therefore predicts that the illusion should be experienced when the observer is standing just in front of the lighthouse, but our account predicts that the illusion only begins to emerge at a critical point roughly 67 m in front of the lighthouse.

Although our primary interest is in the phantom lighthouse illusion, our approach also captures a second previously-reported illusion. Lighthouse beams are typically near parallel to the ground to allow them to travel as far as possible, but they are often perceived to bend down towards the ground (Floor, 1982; Minnaert, 1993). Our theory suggests that the illusion arises from the same kind of distance underestimation that gives rise to the phantom lighthouse illusion. The theory also predicts a novel variant of the bent beam illusion, suggesting that a static beam at constant depth should be perceived as curving towards the observer.

Our Bayesian approach builds on two related approaches from the literature. The first treats perception as a kind of Bayesian inference (Jacobs & Kruschke, 2011; Kersten & Yuille, 2003; Knill & Richards, 1996), and the second proposes that perception is well adapted to the statistical structure of natural scenes (Brady & Field, 2000; Brunswik & Kamiya, 1953; Geisler, 2008; Purves & Lotto, 2003). Our approach is related most closely to the work of Yang and Purves (2003), who used a laser range-finder to construct an empirical prior on distances to the nearest surface at a range of elevation angles. Yang and Purves (2003) show that the resulting prior can account for a family of visual illusions, and the following sections demonstrate that a similar prior accounts for the two lighthouse illusions considered here.

## BAYESIAN PERCEPTION OF VISUAL SPACE

Figure 2a shows an observer in a completely dark environment who is presented with a light at elevation angle *θ*. The observer is uncertain about the distance *d* at which the light lies, but we assume for simplicity that the elevation angle *θ* is perceived accurately and is therefore known. This assumption is consistent with prior empirical work suggesting that perception of *θ* is accurate in the absence of reliable distance cues (Hershenson, 1998; Rock & McDermott, 1964).

**Figure 2.**
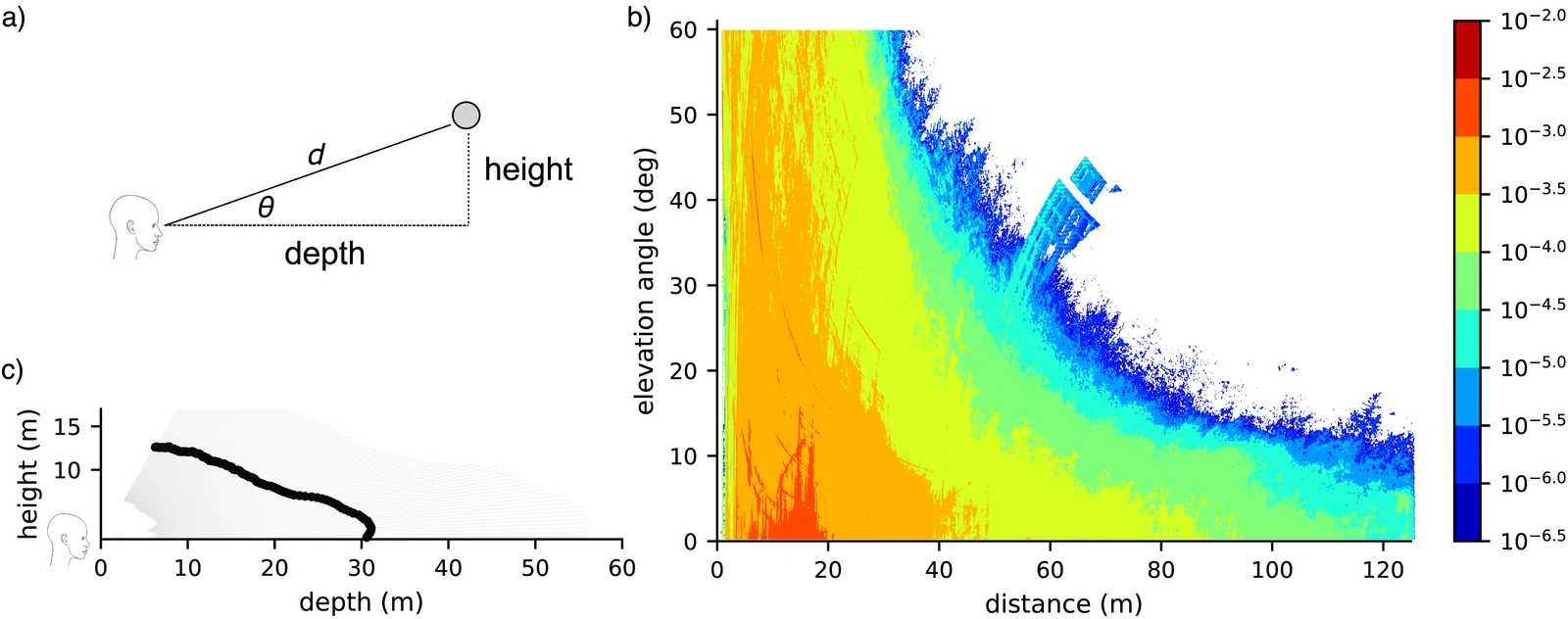
Distance perception and the SYNS prior. (a) An observer in a dark room sees a light at elevation angle *θ* and must infer the distance *d* to the light. A Bayesian observer computes a posterior distribution over *d* by combining a prior over distances with a noisy estimate of the distance of the light. (b) A prior on distances at different elevation angles derived from the 76 outdoor scenes in the SYNS data set. (c) Mean distance (black dots) and standard deviation (grey error bars) at each elevation angle based on the SYNS prior. The y-axis measures heights above the observer’s eye which is assumed to be 1.65 m above the ground, the height of the scanner used to collect the SYNS data.

Suppose that the observer’s visual system provides her with an initial, noisy estimate d* that will be combined with prior expectations to estimate a posterior distribution *P*(*d*|d*, *θ*) over the distance *d*. A Bayesian observer (Jacobs & Kruschke, 2011; Kersten & Yuille, 2003; Knill & Richards, 1996) computes this posterior by combining a prior *P*(*d*|*θ*) and a likelihood *P*(d*|*d*, *θ*):

$$P\left(d|d^{*},\theta \right)\propto P\left(d^{*}|d,\theta \right)P\left(d|\theta \right).$$ (1)

Given the posterior *P*(*d*|d*, *θ*), we assume that the observer relies on an estimator function to generate a point prediction about the distance *d*. To instantiate our Bayesian framework we must therefore specify three elements: the prior, the likelihood, and the estimator. The rest of this section considers two possible choices for each element.

The prior *P*(*d*|*θ*) is assumed to be consistent with the observer’s previous experience of natural environments. Yang and Purves (2003) constructed a prior of this kind by taking a LiDAR scanner into a series of natural environments and repeatedly measuring the distance to the nearest object at a range of different elevation angles *θ*. Morin-Duchesne and Langer (2022, see also Duchesne, 2021) compared this prior to an alternative constructed from LiDAR data for 76 outdoor scenes included in the Southampton-York Natural Scenes (SYNS) data set (Adams et al., 2016).^2^ Our primary analyses use the SYNS prior because the angular range of the Yang and Purves prior is limited to a maximum elevation angle of 36°, and therefore does not cover the superior visual field. Morin-Duchesne and Langer (2022) show that the SYNS prior is qualitatively similar to the Yang and Purves prior, and we report results for the Yang and Purves prior in the Supplementary Information.

The 76 scenes used to create the SYNS prior include both natural scenes (e.g., forests and wetlands) and outdoor scenes from urban areas such as shopping districts and residential areas. The LiDAR scanner used for this data set had a height of 1.65 m and a maximum range of 120 m, and data at elevation angles between −45 and 90 were recorded at an angular resolution of 0.036°. We filtered the data to include only angles between 0 and 60°, which roughly corresponds to the extent of the superior visual field. Figure 2b plots the prior density at each elevation angle, and Figure 2c shows the expected distance to the nearest surface as a function of elevation angle. Consistent with the results of Yang and Purves (2003), the SYNS prior indicates that distances to the nearest object tend to be greatest directly along the line of sight (*θ* = 0), and smoothly decrease as *θ* increases.

The likelihood *P*(d*|*d*, *θ*) captures how probable the noisy estimate d* would be if the true distance were *d*. Here we adopt an extreme approach and assume that the likelihood function is constant, or so flat that it is effectively constant. This assumption is appropriate for perception under conditions so impoverished that the noisy estimate provides no real information about *d*. For example, an observer gazing out over a featureless ocean at midnight may find it impossible to distinguish between a small light that is close (small *d*) and a large light that is distant (large *d*). Although our primary results in this paper are based on a constant likelihood function, we also consider a lognormal likelihood which assumes that the logarithm of the noisy estimate d* is drawn from a Gaussian distribution centered on the logarithm log(*d*) of the true distance:

$$\log\left(d,*\right)∼𝒩\left(\log\left(d\right),\sigma^{2}\right)$$ (2)

This likelihood captures the idea that estimates d* for distant objects are noisier than those for nearby objects. We set the standard deviation *σ* of the Gaussian to 0.044, which matches the Weber fraction reported by Lappin et al. (2006) based on an experimental study of visual distance perception.^3^ For this value of *σ*, the log-normal distribution on d* has a standard deviation of around 0.4 m when *d* = 10, and around 4.4 m when *d* = 100.

The third and final element is the estimator used by the observer to convert a posterior distribution into a single depth percept. Our primary results assume that the depth perceived by the observer is the posterior mean, but the Supplementary Information follows Yang and Purves and reports results for a maximum local mass (MLM) estimator. A maximum a posteriori (MAP) estimator would choose the depth with maximum posterior probability, and the MLM approach relaxes this approach by integrating the posterior probability in the local region around each candidate depth.

Unless otherwise specified, the main text assumes that the prior is the SYNS prior, the likelihood is uniform, and the estimator is the posterior mean. Regardless of which prior, likelihood and estimator we choose from the alternatives described above, however, we find that a Bayesian observer is susceptible to both the phantom lighthouse and bent beam illusions. We start with the bent beam illusion, the simpler of the two.

## THE BENT BEAM ILLUSION

Suppose that an observer with an eye height of 1.65 m is standing with her back against a lighthouse and that a single, static lighthouse beam with a height of 30 m is running over her head. On a dark night no reliable depth cues will be available and the observer’s inference about the distance of any given point along the light beam will be based entirely on her prior. The fact that the curve in Figure 2c bends down towards the ground therefore implies that the observer will perceive the lighthouse beam to have a similar shape.

To confirm this result we enumerated points along the lighthouse beam (blue dots in Figure 3a) and used Equation 1 and the prior in Figure 2b to compute the inferred distances of each of these points (red dots in Figure 3a). In reality a lighthouse beam spreads out as it moves away from the source, but we assume for simplicity that the lighthouse beam is a line. Because we are using a constant likelihood function, the shape of the inferred lighthouse beam in Figure 3a (red dots) is identical to the shape of the prior mean in Figure 2c. Results for other combinations of prior, likelihood and estimator are reported in the Supplementary Information, and in all cases the perceived beam bends towards the ground.

**Figure 3.**
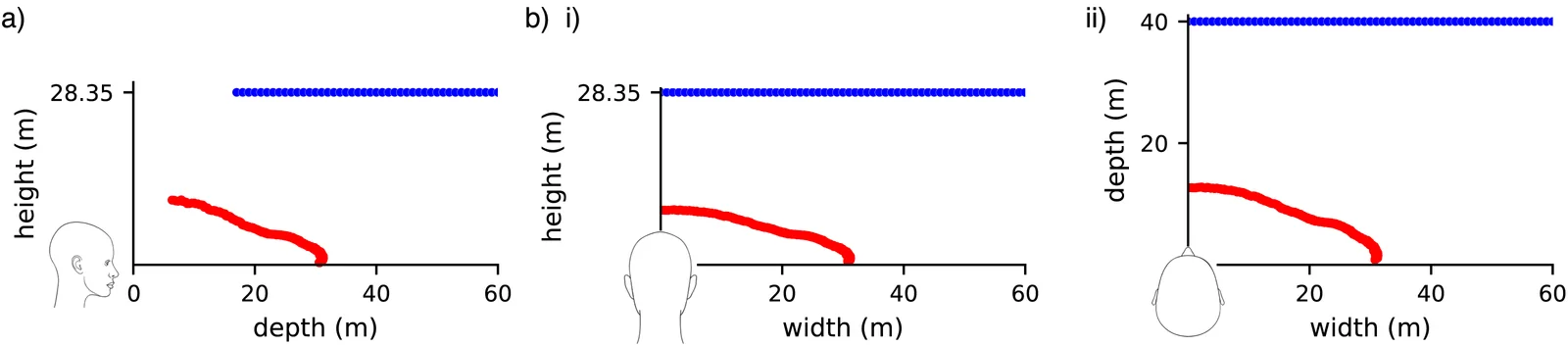
Bayesian inference and the bent beam illusion. (a) Inferred beam (red dots) perceived by an observer who is looking along a horizontal lighthouse beam (blue dots). (b) A Bayesian observer infers that a horizontal beam viewed from the side is bent in two respects. (i) View from behind the observer showing that the beam is inferred to bend towards the ground. (ii) A bird’s eye view showing that the beam is inferred to bend towards the observer. All three panels use the SYNS prior, a constant likelihood function, and the posterior mean estimator.

The result in Figure 3a assumes that the light beam passes directly over the observer’s head, but the model predicts that the bent beam illusion occurs regardless of the observer’s position with respect to the lighthouse. Figure 3b.i shows that a horizontal beam viewed from the side is perceived to bend towards the ground as width (i.e., distance to the observer’s right) increases. Although the beam has constant depth, the bird’s-eye view in Figure 3b.ii shows that the beam is also perceived to bend towards the observer. To our knowledge this depth variant of the bent beam illusion has not been discussed elsewhere, and therefore constitutes a novel prediction of our theory.

## THE PHANTOM LIGHTHOUSE ILLUSION

We now show how distance estimates computed using the prior in Figure 2b can lead to the phantom lighthouse illusion. Figure 4a provides a bird’s eye view of the configuration we consider. Consider an observer located at point *O* who is standing with her back to a lighthouse at *L*. The separation between the observer and the lighthouse is denoted by *s*. The lighthouse beam initially lies along the segment *LB*, but a moment later the beam has rotated and now lies along the segment *LB*′. Here we focus on the solid blue segments of the two lighthouse beams (*AB* and *A*′*B*′). The red segments (*CD* and *C*′*D*′) show how these blue segments are perceived by the observer. Consistent with the prior in Figure 2b, the observer underestimates the distance of each point along the beam, and the discrepancy between inferred distance and true distance increases as the true distance increases. As a result, the two inferred lighthouse beams shown in red converge in a way that suggests a common origin in a phantom lighthouse located at *P*.

**Figure 4.**
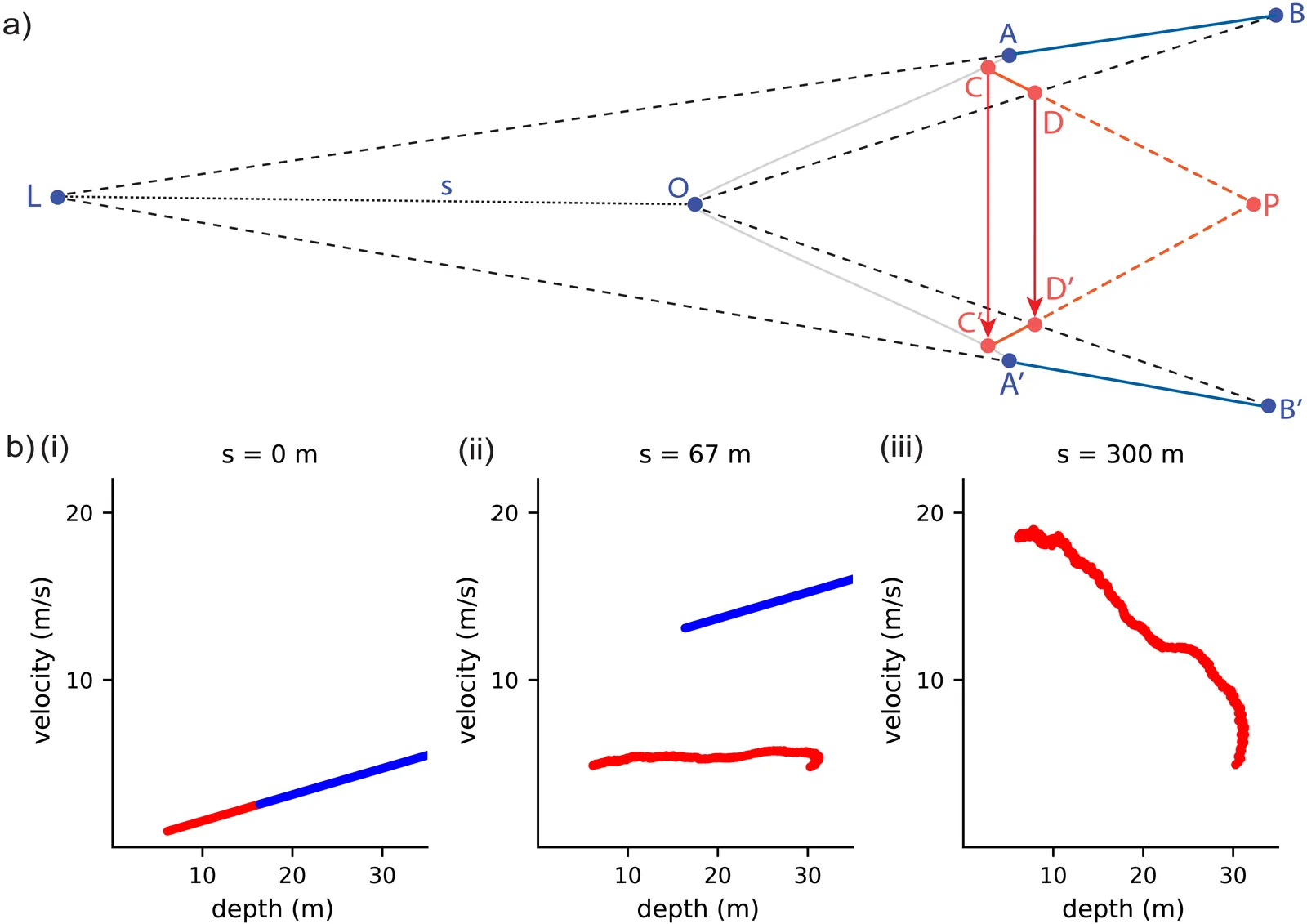
Bayesian inference and the phantom lighthouse illusion. (a) As sketched in Figure 1a, inaccurate estimates of distance can mean that two beams which in reality converge behind an observer are perceived to converge at a point in front of the observer. Here *L* and *O* are the locations of lighthouse and observer, and *AB* is a segment of the true lighthouse beam that rotates to become *A*′*B*′ in the second snapshot of the beam. Bayesian depth estimates mean that segments *AB* and *A*′*B*′ are perceived as segments *CD* and *C*′*D*′, and these two inferred segments converge at point *P*, the location of a perceived phantom lighthouse. (b) Estimated velocities and depths (red dots) and true velocities and depths (blue dots) for different values of *s*, the separation between the observer and the lighthouse. All results assume that the lighthouse has a height of 30 m and takes 40 seconds to complete a full rotation. When *s* is 0 m, more distant points along the beam are perceived as moving faster, consistent with the inference that the source of the beam is behind the observer (no phantom illusion). When *s* is 300 m, more distant points along the beam are perceived as moving slower, consistent with the inference that the beam originates at a phantom lighthouse in front of the observer. The transition between these two percepts occurs at a critical point near *s* = 67 m: at this location, the most distant point on the beam is perceived as having roughly the same velocity as the closest point. In all cases, true velocities increase linearly with depth. In (i) and (ii), the blue curves extend beyond the plot limits, and true velocities and depths (blue dots) are shown only for the closest points on the beam. In (iii), the closest point has a true velocity of around 50 m/s. Videos for *s* = 0 m, *s* = 67 m, and *s* = 300 m are provided in the Supplementary Information.

Although Figure 4a shows the inferred beams converging at a specific point, we do not assume that the observer can estimate this point with any precision. Instead, our approach requires only that the observer estimate the relative velocities of different points along the beam, and we propose that the slower end of the beam is inferred to be the source. This proposal is broadly compatible with previous evidence for “slow-and-smooth” priors in motion perception (Weiss & Adelson, 1998; Weiss et al., 2002; Yuille & Grzywacz, 1988), and suggests that the phantom illusion occurs only if the most distant points along the beam (e.g., point *D* in Figure 4a) are inferred to have lower velocities than the closest points (e.g., point *C* in Figure 4a). In Figure 4b, the red arrows show velocity estimates, and the reduced length of the arrow for *D* relative to the arrow for *C* indicates that the observer at *O* experiences the illusion. In the experience of the first author, the spurious source of the beams is sometimes perceived as moving rather than stationary, as if the phantom lighthouse were carried by a speeding boat far out at sea. Characterizing the illusion in terms of the relative velocities of close and distant points ensures that percepts involving a moving source still qualify as instances of the illusion.

Whether or not the illusion is experienced depends on the separation *s* between the observer and the lighthouse. When the separation is 0 m (ie the observer is standing at the base of the lighthouse), Figure 4b.i shows that inferred velocities increase monotonically with depth, which means that no illusion is predicted to occur. When the separation is 300 m, the inferred velocities for the most distant points are substantially lower than the inferred velocities for the nearest points along the beam, suggesting that a strong illusion is experienced. Figure 4b.ii suggests that the critical point marking the transition between these two regimes is predicted to occur for separations *s* around 67 metres. At this critical separation the inferred velocities of the most distant points match the inferred velocities of the closest points, which suggests that the observer may be uncertain whether the source lies in front of her or behind her.

We identified the critical separation by computing inferred beams for all integer values of *s* starting at *s* = 0 m, and identifying the first case in which the inferred velocity of the most distant point along the beam was smaller than the inferred velocity of the closest point along the beam. For a 30 m lighthouse, the crossover point occurs at a separation of *s* = 67 m, and for lighthouse heights of 34 m and 38 m (the elevation of the Lady Elliot lighthouse) the crossover points occur at separations of *s* = 78 m and *s* = 89 m, respectively.^4^

If we replace the uniform likelihood with the log-normal likelihood, the critical separation for a 30 m lighthouse is 120 m, and if we replace the posterior mean with the MLM estimator, the critical separation is 54 m. Results for other combinations of prior, likelihood and estimator are reported in the Supplementary Information. Different combinations lead to different numerical predictions, but overall our theory predicts that the phantom illusion emerges at a critical separation on the order of 100 m. Previous scholars report experiencing the phantom lighthouse illusion at separations of 1 km (Colange & Le Grand, 1937) and 4 km (Dunoyer, 1937), but we know of no previous reports that document the illusion at separations closer to 100 m. Our theory therefore implies that the illusion can be experienced at separations an order of magnitude smaller than those previously reported.^5^

We developed a virtual reality experiment using a 30 m lighthouse to test the categorical prediction that a critical point exists, and the quantitative prediction that this point occurs at a value of *s* around 67 metres. Although our primary focus is the phantom lighthouse illusion, our experiment also explores the bent beam illusion, which provides direct evidence about the phenomenon of distance underestimation that is proposed to drive the phantom illusion.

## EXPERIMENT: LIGHTHOUSE ILLUSIONS IN VIRTUAL REALITY

The experiment was carried out with approval from the Human Ethics Committee at the University of Melbourne, and included three conditions summarized in Table 1. The phantom condition used a rotating beam and aimed to estimate the critical separation *s* at which the phantom lighthouse illusion begins to occur. The height condition used a stationary beam viewed from the side, and aimed to estimate the elevation angle (or the angle with respect to the horizontal plane) at which the beam is perceived as having constant height. If a horizontal beam is perceived as bending towards the ground (Figure 3b.i), then the beam would need to be tilted up (positive elevation angle) in order to be perceived as having constant height. The depth condition was directly analogous to the height condition, and aimed to estimate the azimuth angle (or the angle of the beam within a horizontal plane) at which the beam is perceived as having constant depth. If a beam viewed from the side is perceived as bending towards the observer (Figure 3b.ii), then the beam would need to be rotated away from the observer in order to be perceived as having constant depth.

**Table 1.** Summary of the three experimental conditions. We refer to the estimand in the height condition as a threshold because it corresponds to the angle that must be exceeded in order for the beam to be perceived as pointing up. Similarly, the estimand for the depth condition can also be viewed as a threshold.

**Condition**	**Estimand**
phantom	critical separation (separation between observer and lighthouse at which the phantom illusion emerges)
height	elevation threshold (elevation angle at which the beam is perceived as having constant height)
depth	azimuth threshold (azimuth angle at which the beam is perceived as having constant depth)

We used both a staircase method and the method of adjustment to estimate the three variables of interest (critical separation, elevation angle, and azimuth angle). The experiment was run entirely within participants, so each participant completed both staircase and adjustment phases for all three conditions.

The experiment was preregistered, but at the time of designing the experiment and collecting the data we did not have access to the SYNS prior or the Yang and Purves prior. Our predictions were therefore derived from a prior based on synthetic scenes in which rectangular surfaces of different sizes were placed at different distances from the observer (Xu et al., 2023). Based on this synthetic prior we expected a critical separation around 75 m, and preregistered the hypothesis that “the phantom lighthouse illusion will begin to emerge when the distance between viewer and lighthouse is between 50 and 100 m.”

### Participants

31 participants completed the experiment in exchange for course credit at the University of Melbourne. There was an error in our initial implementation of the depth staircase that affected the first 7 participants, so we dropped all data from the depth condition for these participants. The preregistration indicated that we would recruit 24 participants and we met this goal for the depth condition and exceeded it for the phantom and height conditions.

The preregistration also indicated that participants with a total response time more than 3 standard deviations away from the group mean would be excluded. One participant was excluded on this basis leaving 30 participants in the phantom and height conditions and 23 in the depth condition.

### Materials

The experiment used a custom-built interface that was developed in Unity and deployed on a Meta Quest 2 headset. This headset has a weight of 503 g and horizontal and vertical fields of view of 104 and 98°. The scene for each condition included a narrow white cylinder (the lighthouse beam) in a completely dark environment illuminated by a single spotlight. The section of the cylinder that fell within the light cast by the spotlight was visible, and the rest of the cylinder was invisible. Screenshots of the phantom and height conditions are shown in Figures 5a and 5b, and screen recordings of the phantom condition are available as Supplementary Movies S1, S2, and S3.

**Figure 5.**
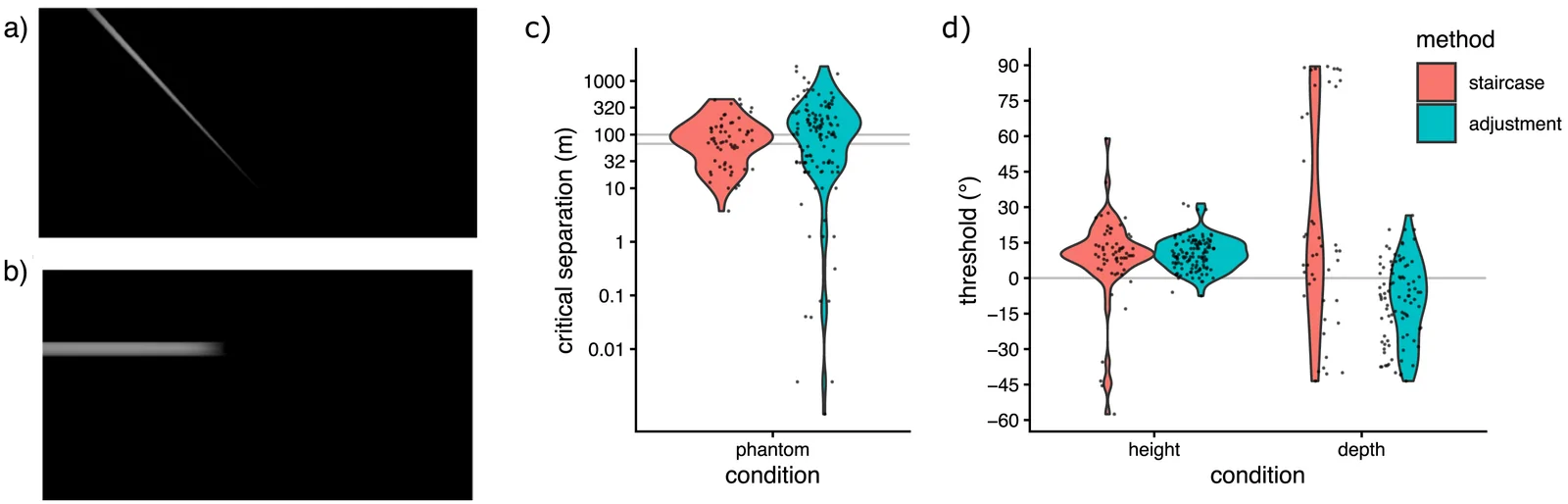
Virtual reality experiment. (a) Screenshot of the phantom condition. Supplementary Movies S1, S2, and S3 are screen recordings of this condition. (b) Screenshot of the height condition. The depth condition looked very similar to the height condition (c, d) Staircase and adjustment results for the phantom, height and depth conditions. Except for exclusions mentioned in the text, each pair of violins includes two staircase datapoints and four adjustment datapoints for each participant. In (c), horizontal grey lines show critical separations of 67 (model prediction) and 100 (pre-registered upper bound on the average critical separation).

Rendering light beams in virtual reality is a notorious challenge, and the beams in Experiment 2 could not be mistaken for genuine beams of light—they appeared solid rather than ethereal, and their edges were too sharp. We experimented with a volumetric light beam (Tech Salad, 2023) but found that a cylinder illuminated by a spotlight looked about as realistic and offered two additional advantages. In the phantom condition, the spotlight illuminated only part of the rotating beam, which meant that participants could not see the source of the beam if they turned around and looked behind their backs. In the height and depth conditions, the beam started in the middle of the scene and extended off to the left, and the spotlight approach meant that the rightmost end of the beam appeared to grade off into darkness instead of terminating abruptly.

In reality, a lighthouse beam spreads out as it moves away from its source, and we therefore considered using a conical beam instead of a cylinder. We settled on a cylinder because this approach seemed simpler and we felt that it would give us the best chance of observing the predicted effects in the height and depth conditions. In the height condition, perceiving whether a cylinder is horizontal seems easier than perceiving whether the axis of a cone is horizontal. In the depth condition, the apparent tapering of a cylindrical beam provides a cue that can potentially be used to detect whether or not a beam has constant depth.

In addition to scenes for the three conditions, the experiment included a demo scene that showed two white spheres, again illuminated by a single spotlight, and required participants to assess the relative sizes of the two spheres. The right sphere always had a radius of 1 m, and the radius of the left sphere varied based on participants’ responses. The variable estimated for this scene was the size ratio, or the diameter of the left sphere divided by the diameter of the right sphere. The purpose of this demo was to familiarize participants with the kinds of questions asked during the three conditions.

In all three conditions the diameter of the cylindrical lighthouse beam was 1 m. In the phantom condition, the source of the beam was located behind the user (i.e., in the direction opposite to the initial gaze direction) at a height of 30 m. In the height and depth conditions, the beam was anchored on a point in the centre of the initial field of view with a height of 30 m and a depth of 25 m.

The experiment included two staircases for each scene, and these staircases asked participants to judge which sphere looked bigger (demo), whether the source of the beam was behind them or in front of them (phantom), whether the beam was tilted up or tilted down (height) and whether the beam was tilted towards them or away from them (depth). The demo staircases included four reversals, and each step adjusted the right sphere in order to increase the size ratio by $\frac{11}{10}$ or reduce it by $\frac{10}{11}$. The two staircases were initialized at size ratios of $\frac{2}{3}$ and $\frac{3}{2}$. The phantom height and depth staircases included six reversals and the step size halved after the first and third reversals. The initial step sizes were 20 m for the phantom condition and 4 degrees for the height and depth conditions. The staircases were initialized at separations of 30 and 150 m (phantom condition) and angles of 10 and −10 degrees (height and depth conditions).

The experiment included 4 adjustment phases for each scene. The adjustment phases were initialized using the same values as the staircases, and the step sizes were 5 m (phantom condition) and 2.5 degrees (height and depth conditions). For the phantom scene, participants were asked to adjust the scene (i.e., increase or decrease the separation between them and the lighthouse) until the beam looked as if it were sweeping horizontally over their heads. We chose this question because it asked about a perceptual feature that could be identified with confidence. In contrast, asking about the beam’s source would have required participants to generate a stimulus associated with uncertainty—specifically, to adjust the beam until they were unsure whether the source was in front of them or behind them. For the remaining scenes the adjustment instructions corresponded closely to the staircase instructions, and participants were asked to adjust the scene until the spheres looked identical in size (demo), until the beam looked horizontal (height) or until the beam looked parallel to the middle line at their feet, and neither tilted towards them or away from them (depth). The depth question referred to a guide located on the floor of the VR environment that showed a line tilting towards the participant, a line at constant depth, and a line tilting away from the participant. In the height condition a similar guide located just in front of each participant’s waist showed a line tilted up, a line tilted down, and a horizontal line. We included these guides to minimize uncertainty about spatial descriptors such as “tilting up” and “tilting away.” For example, a beam that extends from the bottom right of the visual field to the top left is “tilting up” relative to the right endpoint of the beam but “tilting down” relative to the left endpoint.

### Procedure

The order of the phantom, height and depth scenes was randomized across participants. All participants completed two staircases for the demo scene and then two staircases for each of the remaining scenes before moving on to the adjustment phase. The staircases were always presented first because in the phantom condition only the staircase instructions explicitly asked about the source of the lightbeams. We therefore viewed the staircase method as the most accurate way to estimate the critical separation, and wanted the staircase results to be uninfluenced by previous exposure to the adjustment task.

### Results

For each condition, we have two staircase estimates and four adjustment estimates per participant, and all estimates are plotted in Figure 5c. The estimate for each staircase is defined as the average of the final two reversals.

In the phantom condition, the median critical separations are 75 m (staircase method) and 112.5 m (adjustment method). As mentioned already, we preregistered the hypothesis that the “phantom lighthouse illusion will begin to emerge when the distance between viewer and lighthouse is between 50 and 100 m.” The median for the adjustment method falls outside this range, but not by much, and the two methods provide converging evidence that the critical separation is not far from the theoretical prediction of 67 m.

For the adjustment method, 6 participants generated at least one response smaller than 1, and there are 8 of these responses in total. It is possible that these small responses are an artifact of our VR experiment. To ensure that participants remained in front of the true source of the beam at all times, whenever a staircase or adjustment step would have left the distance between participant and the source less than 1 m, we replaced this step with a special-purpose step that halved the current distance between the participant and the source. Responses less than 1 may therefore not indicate that a participant actually saw the illusion at a critical separation less than 1 m, and may have instead been produced by participants who were exploring the limits of the display (i.e., pressing one button repeatedly in the adjustment phase to see how the scene would change).

We computed posterior distributions separately for the staircase and adjustment data using lognormal mixed-effects models with critical separation as the dependent variable and a random effect for participant. All models used default priors provided by the brms package in R (Bürkner, 2017). The 95% credible intervals on the critical separation were [51 m, 95 m] (staircase data), [31 m, 104 m] (adjustment data including responses smaller than 1 m) and [67 m, 153 m] (adjustment data without responses less than 1 m). All three intervals include 67 m, the theoretical prediction. Taken together, the data for the phantom condition suggest that the phantom illusion is experienced in VR, that the illusion begins to emerge at a critical separation, and that this critical separation is around 67–95 m. Our data therefore confirm the prediction that the illusion occurs at separations an order of magnitude smaller than the separations (1 km and 4 km) previously reported in the literature (Colange & Le Grand, 1937; Dunoyer, 1937).

In the height condition, Figure 5d suggests that the beam needed to be pointing up in order for participants to see it as horizontal. Median threshold angles were 10 and 9.75 degrees above the horizontal for the staircase and adjustment methods respectively. We computed posterior distributions on the thresholds using linear mixed-effects models with threshold angle as the dependent variable and a random effect for participant, and the 95% credible intervals on the thresholds were [4.4°, 13.4°] (staircase) and [7.8°, 12.3°] (adjustment). In both cases the 95% credible interval excludes zero, suggesting that a beam does have to be tilted above the horizontal in order to be perceived as having constant height.

In the depth condition, the staircase data suggest that a beam has to be tilted away from the participant (median angle of 11.5°) in order to appear of constant depth, but the adjustment data suggest the opposite (median angle is −7.8°). The Supplementary Information describes some additional reasons to think that these results are not reliable. Our experiment does suggest, however, that participants found the depth condition relatively difficult, and that the depth illusion (if it exists) is weaker and more variable than the other two illusions in our experiments (horizontal bent beam and phantom lighthouse).

## DISCUSSION

We presented and tested a formal theory which suggests that the phantom lighthouse illusion occurs because observers use Bayesian inference to infer the distance of points along a lighthouse beam. Our theory suggests that the illusion depends on the observer’s prior distribution over distances, and we used a prior derived from natural scene statistics. The prior incorporates no free parameters, and leads to the prediction that the phantom lighthouse illusion emerges at a critical point where the separation between the observer and the lighthouse is around 67 m.

Our experiment demonstrated that the phantom illusion can be experienced in virtual reality, and the staircase method yielded a 95% credible interval of [51 m, 95 m] on the critical separation. The experiment also explored the bent beam illusion, and we found that a beam needs to be tilted roughly 10 degrees above the horizontal in order to appear of constant height. We did not find support, however, for the prediction that a beam of constant depth is perceived as tilting towards the observer.

### Challenges and Open Questions

We tested the depth version of the bent beam illusion in VR and the natural next step is to test the illusion in the real world using a static horizontal beam. Goldstein (1966) reports that a searchlight tilted away from the observer is perceived as tilting towards the observer, but to our knowledge no previous study considers the perception of horizontal light beams of constant depth. If a careful real-world study finds no evidence that beams of constant depth are perceived as tilting towards the observer, then our Bayesian model would be seriously challenged.

Our theoretical approach assumed for simplicity that a lighthouse beam is a line of points or an infinitely thin cylinder, but a real-world lighthouse beam is a cone rather than a cylinder, and the shape of this beam may contribute to both the bent-beam and phantom illusions. If the axis of a conical beam is horizontal then the bottom surface of the cone tilts down the ground, and the bent beam illusion could therefore arise if observers are tracking the bottom of the conical beam rather than its center. A conical beam could also contribute to the phantom illusion if observers rely on the prior expectation that the source of the beam is likely to be low to the ground. This expectation suggests that an inclined surface such as the bottom of a conical beam is more likely to be generated by a low light source pointing up than a high light source pointing down, which could give rise to the phantom illusion. Future VR studies could manipulate the shape of the beam to explore the extent to which the illusions are sensitive to this factor. Our experimental results, however, suggest that a cylindrical beam is enough to produce both illusions, which implies that beam shape may not be a critical factor.

Any Bayesian theory relies on a prior and a likelihood function, and both components deserve further investigation. We assumed that the prior is consistent with natural scene statistics, but our theory is agnostic about the origin of this prior. One possibility is that the prior is learned from previous experience, but it is also possible that this “learning” took place over the course of evolution and that the observer is innately provided with the prior. If the prior is learned from experience, then this learning must take place in conditions under which the observer has relatively reliable information about the true distances of perceived objects. Sedgwick (1986, p. 21.9) notes that the perception of distance is quite good when an object “is viewed in an illuminated, structured and textured environment with binocular vision and at least some freedom of head movement,” which makes it plausible that an observer could learn an empirical prior that comes close to the LiDAR based prior used in our analyses.

For simplicity, our primary analyses assumed that the likelihood function is constant which means that it makes no contribution to our results. This assumption seems appropriate for an observer viewing lighthouse beams in a dark and featureless environment, but will need to be adjusted for situations in which reliable cues to distance are available. Adding reliable depth cues may change the shape of the inferred beam, and depth cues will also affect the critical separation associated with the phantom lighthouse illusion.

Following previous work (Landy et al., 2011; Yang & Purves, 2003), the alternative lognormal likelihood function we considered was formulated using a noisy but unbiased estimate d* of the true depth. This noisy estimate is sometimes called *registered distance* (Epstein, 1973; Hershenson, 1998; Kaufman & Rock, 1989), and must be distinguished from the *inferred distance* that is computed by combining the registered distance with the prior. Because registered distance cannot be directly measured in the psychological laboratory, its theoretical status is controversial, and there is no consensus about how it may be computed and represented. We have mostly avoided this debate by focusing on a constant likelihood function, but future applications of our theory that focus on alternative likelihood functions will need to engage in the debate more directly.

Although we have not attempted to model individual differences, our empirical results in Figure 5 show substantial variation across individuals. One possible explanation consistent with our Bayesian account is that different observers rely on different priors, and perhaps also different likelihood functions. The variability we observed is consistent with previous work suggesting that the variability of distance estimates under reduced viewing conditions is high (Sedgwick, 1986). For example, Landauer and Epstein (1969) developed an experiment in which observers surrounded by darkness reported the distance of a dimly illuminated disc located up to a few metres in front of them, and report that several participants gave astronomical estimates (e.g., “the same distance as the moon.”)

### Theoretical Accounts

The prediction of a critical point distinguishes our theory from the *naive projection* account mentioned in the introduction (Floor, 1982; Minnaert, 1993). This alternative account assumes that the observer takes retinal distance as a proxy for distance in the real world, and suggests that the phantom illusion occurs because projections of successive snapshots of the beam converge within the retinal plane. The simplest version of this theory predicts that the phantom lighthouse illusion should be experienced for all values of *s* > 0 m. For example, the projection in Figure 1b was computed for *s* = 67 m, but when *s* is reduced the three projected beams still converge in the retinal plane as long as *s* > 0 m. The naive projection account is therefore incompatible with all of the 95% credible intervals on the critical separation yielded by analyses of our experimental data.

Our theory proposes that the observer infers the source of the beam by comparing inferred velocities of near and distant points along the beam. An alternative approach might propose that the relevant comparison involves retinal speeds rather than inferred real-world velocities. This alternative turns out to be equivalent to the naive projection account, because the retinal projections of distant parts of the beam move slower than the projections of near points only if the projected beams converge in the retinal plane.

We believe that the existence of a critical point is the greatest challenge for the naive projection account and the equivalent account based on retinal speed. These accounts, however, also fail to account for our experimental finding that a horizontal beam is perceived as tilting towards the ground. In contrast, we showed that a Bayesian account of distance perception captures both the bent beam illusion and the finding that the phantom illusion only emerges when the observer is situated at some distance in front of the lighthouse.

Although we have treated the naive projection account as a competitor to our theory, it is possible that underestimation of distance (the focus of our theory) and inadequate allowance for perspective (the focus of the naive projection model) both contribute to the phantom illusion. Other factors may also play a role, including photometric brightness (Colange & Le Grand, 1937), expectations about speed (Stocker & Simoncelli, 2006; Weiss et al., 2002) and other factors related to motion perception. Future theoretical and empirical work can therefore aim to develop and test combined approaches that draw on two or more of these factors.

## CONCLUSION

We developed a Bayesian account of the phantom lighthouse illusion and presented evidence supporting the key prediction that the illusion begins to emerge when the viewer is between 50 and 100 m away from the lighthouse. The phantom illusion has been observed for decades but has received little systematic investigation, and our work suggests that the illusion can be productively studied using computational models and behavioral experiments.

Although the phantom illusion itself is intriguing, its broader significance is to provide a window onto the perception of visual space (Hershenson, 1998). A second real-world illusion with the same significance is the moon illusion, or the observation that the moon appears larger near the horizon than at its zenith (Kaufman & Rock, 1989; Rudrauf et al., 2020). The moon illusion has inspired a large literature that considers different theoretical accounts of perception, and we believe that the phantom lighthouse illusion will repay equally detailed scrutiny.

## ACKNOWLEDGMENTS

We thank Xavier Morin Duchesne for sharing code and data that allowed us to generate the empirical priors used in this project, and Anthony Lua for comments on the manuscript. Lisa Tran helped to develop and run the VR experiment as part of her Honours project in 2023. A preliminary version of this work was presented at the Annual Meeting of the Cognitive Science Society in 2023.

## FUNDING INFORMATION

This work was supported in part by ARC FT190100200.

## AUTHOR CONTRIBUTIONS

C.K.: Conceptualization; Investigation; Methodology; Software; Writing – original draft; Writing – review & editing. W.X.: Software; Writing – original draft. S.J.C.: Conceptualization; Methodology; Writing – review & editing.

## DATA AVAILABILITY STATEMENT

Code, data and experimental materials are available at https://github.com/cskemp/phantomlighthouse.

The preregistration for the experiment is available at https://aspredicted.org/16Y_72N.

## Notes

^1^ When the optical center of a pinhole camera is directly below a rotating lighthouse beacon, each lighthouse beam projects to a vertical line in the image plane and all of these projected beams are parallel. If the optical center is in front of the beacon by even a small amount, the projected beams converge in the image plane as shown in Figure 1b.^2^ Morin-Duchesne and Langer (2022) also consider a third prior derived from the Semantic3D data set (Hackel et al., 2017). Because this data set was collected only in urban settings it seems less suitable for the current project.^3^ Lappin et al. (2006) report Weber fractions for three settings (lobby, hall, and lawn) and two distance levels (15 m and 30 m). We used the result for the lawn at 30 m, because that seemed like the closest match to a setting in which people observe a lighthouse beam that extends far into the distance in a setting with few environmental distance cues. This result, however, almost certainly underestimates the variability involved when people observe a lighthouse beam on a dark night.^4^ All of these results assume that the lighthouse and observer are situated on level ground, and that the height of the observer is 1.65 m. If the base of the lighthouse is higher than the ground on which the observer is standing, then this difference increases the height of the beam relative to the observer, which in turn increases the critical separation. While this manuscript was in press, we realized that our model's predictions about the critical separation also depend on the true depth of the furthest visible part of the beam. Our analyses assume that the beam is visible to a depth of 2,500 m, and increasing this depth to 10,000 m yields a critical separation of 65 m for a 30 m lighthouse.^5^ This prediction is consistent with the personal experience of the first author and several of his family members, who all experienced the illusion on the west shore of Lady Elliot Island at a separation smaller than 90 m.

## Supplementary Material



s0

s67

s300
