# Supplementary material for: A Bayesian Account of the Phantom Lighthouse Illusion
Source: Open Mind (Camb). 2026 Oct 3;10:1593–608. doi: 10.1162/OPMI.a.390 (PMC13733552; doi:10.1162/OPMI.a.390)
Supplement: Supplementary file 1 [file opmi-10-1593-s001.pdf]

Supplementary Information

Charles Kemp, Weilun Xu & Simon J. Cropper

1 Yang and Purves Prior **1**

2 Results for alternative combinations of prior, likelihood and estimator **2**

3 Depth condition of the experiment **3**

4 Preregistered analyses **5**

5 SI Movies **6**

1 Yang and Purves Prior

Yang and Purves compiled a prior over distances at different elevation angles using data from 23 natural scenes and 51 outdoor scenes. The LiDAR scanner used had a height of 1.65 m and a maximum range of 300 m, and data at elevation angles between -45° and 40° were recorded at an angular resolution of 0.144°. Yang and Purves removed the edge of each image leaving a maximum elevation angle of 36°, and we did the same before filtering the data to include only angles between 0° and 36°. Compared to the SYNS prior, the Yang and Purves includes a larger range of distances (the SYNS scanner had a maximum range of 120 m), but the data are lower in resolution and do not cover the full extent of the superior visual field.

Figure S1b plots the prior density at each elevation angle, and Figure S1c shows the expected distance to the nearest surface as a function of elevation angle. Both plots are based on the original version of the Yang and Purves prior, but for subsequent analyses we used a version created by applying smoothing across both angles and distances using a kernel of $[1, 4, 6, 4, 1]$. The original version of the prior is somewhat noisy, which means that smoothing is desirable when estimating critical separations based on this prior.

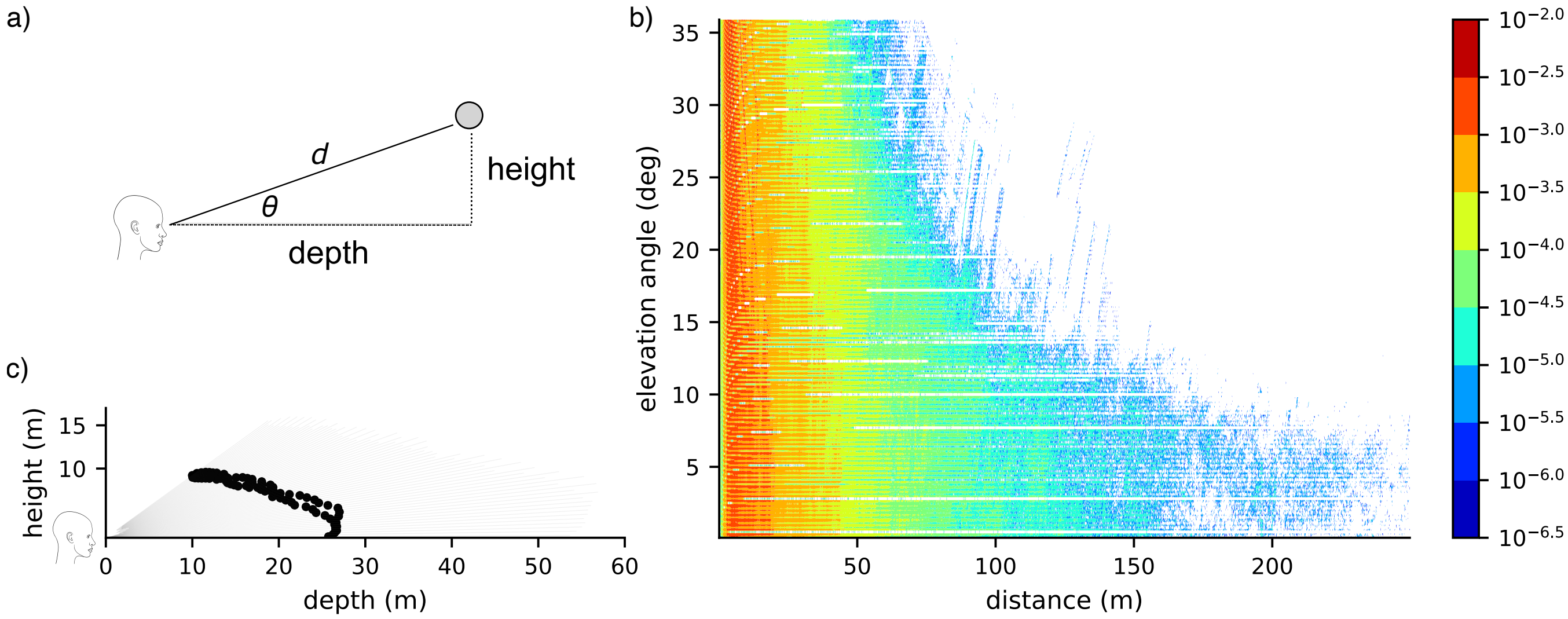

Figure S1: Version of Figure 2 based on the Yang and Purves prior. (a) An observer sees a light at elevation angle θ and must infer the distance d to the light. (b) A prior on distances at different elevation angles compiled by Yang and Purves. (c) Mean distance (black dots) and standard deviation (grey error bars) at each elevation angle based on the Yang and Purves prior.

2 Results for alternative combinations of prior, likelihood and estimator

Our primary results assume that the inferred distance at a given elevation angle is the mean of the posterior distribution, which corresponds to assuming a squared-error loss function. We also considered a maximum local mass estimator (MLM), which uses an inverse Gaussian as the loss function:

$$L(\hat{d}, d) = -\exp\left(-\frac{(\hat{d}-d)^2}{2\sigma_l^2}\right), \tag{S1}$$

where $L(\hat{d}, d)$ is the loss for estimate $\hat{d}$ when the true distance is d, and σ_l captures the spread of the loss function. Following Yang and Purves we set $\sigma_l = 0.2$. This value of σ_l is rather small, and means that the MLM estimator behaves similarly to a MAP estimator in our setting.

Figure S2 shows inferred beams (red dots) for all possible combinations of two priors (SYNS and Yang and Purves), two likelihoods (uniform and lognormal) and two estimators (mean and MLM). Figure S2a is identical to the plot reported in Figure 3a of the main text. In all cases, the inferred beam curves toward the ground, but the inferred beams based on the lognormal likelihood extend to a much greater extent than the beams for the uniform likelihood. This result makes sense because the standard deviation for the lognormal likelihood is relatively small (0.044), which means that the inferred depths of points along the beam track the true depths relatively closely for

the first hundred metres or so.

The results for the mean and MLM estimators are relatively similar when using the lognormal likelihood, but quite different when using the uniform likelihood. When the likelihood is uniform, the MLM approach infers that all points on the beam are relatively close to the observer, because the prior density on distances (Figures 2b and S1b) assigns most probability to small distances. Despite this difference, the critical separations for the mean and MLM estimators are relatively close for the SYNS prior with uniform likelihood (67 m versus 54 m). These separations, however, are very different for the Yang and Purves prior with uniform likelihood (47 m versus 1 m). A critical separation of 1 m matches the prediction of the naive projection account that the phantom illusion is experienced for any separation greater than 0 m.

The two priors (SYNS and Yang and Purves) produce qualitatively similar results when paired with the uniform likelihood and mean estimator, and the lower critical separation for the Yang and Purves prior (47 m vs 67 m) is consistent with the fact that the Yang and Purves result is based on a maximum elevation angle of only 36° rather than 60°. At a separation of 47 m, the Yang and Purves result suggests that the inferred velocity of the point at 36° matches the inferred velocity of the most distant point on the beam — but the inferred velocity would decrease for elevations higher than 36°, meaning that 47 m is an underestimate of the true critical separation. When paired with the lognormal likelihood, the critical separations for the Yang and Purves prior exceed those for the SYNS prior, which presumably occurs because the Yang and Purves prior has support over a much greater range of distances than does the SYNS prior.

Two of the results for the uniform likelihood ($s = 67$, $s = 54$) fall within the credible interval of [51 m, 95 m] derived from our staircase data, and a third ($s = 47$) lies just outside this range. The fourth result for the uniform likelihood ($s = 1$ for Yang and Purves prior and MLM estimator) is inconsistent with our data, but may be unduly influenced by noise in the Yang and Purves prior. Overall, the combination of a uniform likelihood and mean estimator seems most consistent with our experimental data.

3 Depth condition of the experiment

The main text noted that the staircase and adjustment methods produced inconsistent results for the depth condition, and suggested that the data for this condition are unreliable. As described in the main text, the VR scene for this condition included a cylinder (the beam) illuminated by a single spotlight. When designing the depth scene we had anticipated that the angle of the beam would mostly remain within the interval [-30°, 30°], and the scene looked

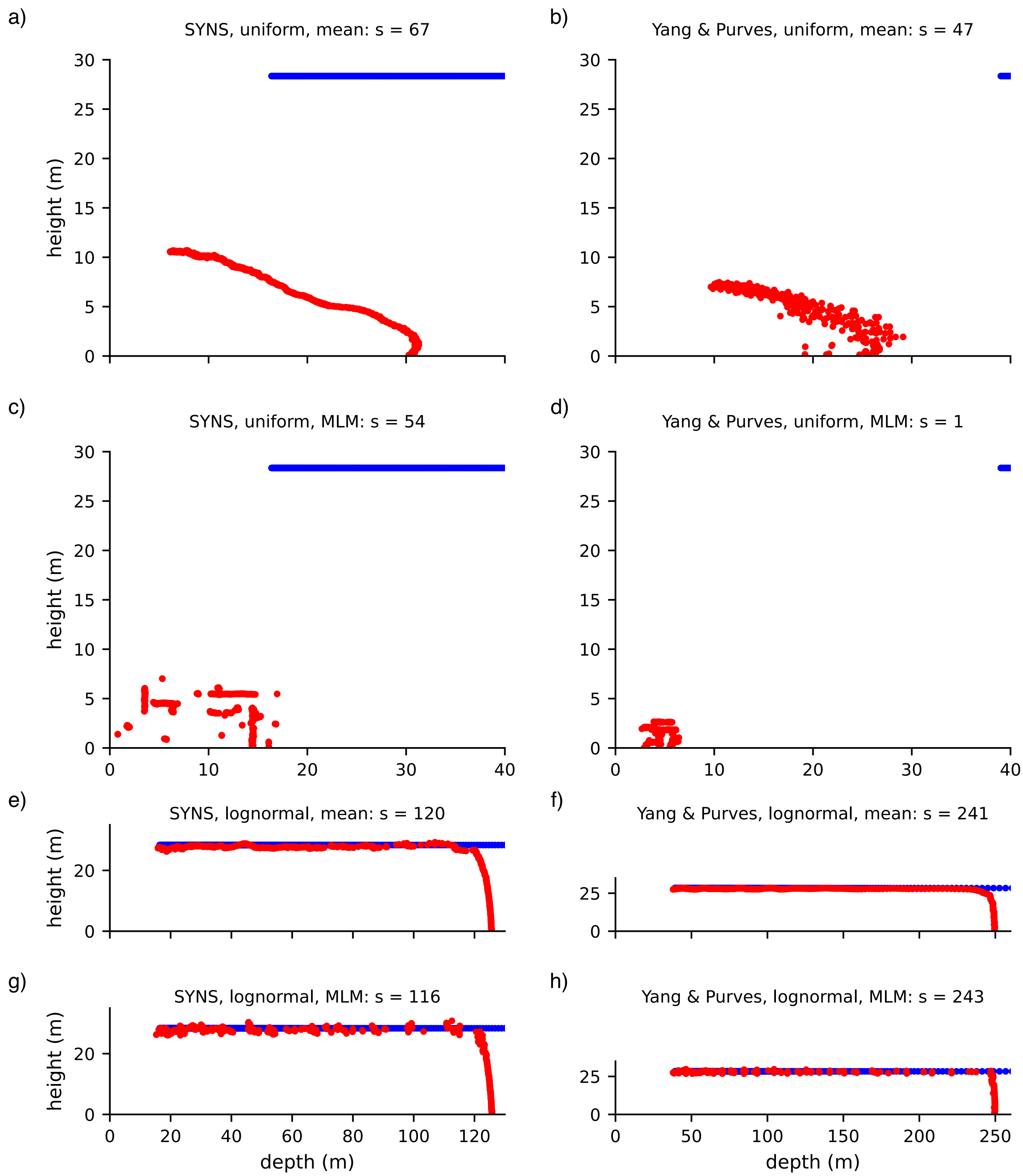

Figure S2: Inferred beams (red dots) for alternative combinations of prior, likelihood and estimator. The label for each panel includes the critical separation s for that combination of prior, likelihood and estimator. Panels (a) and (e) are based on the original SYNS prior, and panels (c) and (g) are based on a smoothed version of this prior. All results in the right column are based on a smoothed version of the Yang & Purves prior. The true lighthouse beam (blue) starts at different depths in the left and right columns because the maximum elevation angles differ across the two priors (60° versus 36°).

reasonable for angles within this range. Outside of this range, however, the scene began to look increasingly strange. For example, when the angle was 90° the cylinder was entirely invisible, and as the angle began to approach 90° the visible portion of the cylinder became shorter and shorter. In the depth condition, multiple participants ended up at angles for which the VR scene looked strange, and around 5 participants had to ask the experimenter for help because the lighthouse beam had apparently disappeared (i.e. because the angle was close to 90°).

Some participants may have had trouble with the depth condition because their interpretation of "pointing towards" and "pointing away" differed from the interpretation we intended. If the beam is anchored on the right (as it actually was), then it is natural to say that a beam is "pointing toward" the participant if the left end has smaller depth than the right depth. The experimenter described this interpretation in the pre-task briefing using a physical prop (a pencil), and it was reinforced in the experiment by the visual guide provided on the floor of the VR scene. If the beam were anchored on the left, however, then it is natural to say that a beam is "pointing away from" the participant if the left end has smaller depth than the right depth. In debriefing one participant mentioned that she had initially followed this interpretation, and changed to the desired interpretation only partway through the task. The undesired interpretation may have been facilitated by the fact that the left side of the beam was further away than the right side and therefore appeared narrower.

A future VR experiment may be able to use a design that alleviates some of these issues. The current experiment, however, does suggest that participants found the depth condition relatively difficult, and that the depth illusion (if it exists) is weaker and more variable than the other two illusions in our experiments (horizontal bent beam and phantom lighthouse). The problems participants had with the depth condition suggest that they did not perceive the stimulus consistently even within the acceptable range of angles — the reason many participants ended up outside this range is presumably because they felt that angles within the acceptable range did not produce a beam that appeared to be of constant depth.

4 Preregistered analyses

The main text reported 95% credibility intervals on the thresholds for the phantom and height conditions. For the depth condition, 95% credible intervals for the threshold angle were [4.3°, 39.1°] (staircase method) and [-15.5°, -3.7°] (adjustment).

The pregistration also mentioned combined models that draw on data from both staircase and adjustment meth-

ods and include a random intercept for method. 95% credible intervals on thresholds obtained using these combined models were [24.3 m, 365 m] (phantom), [5.0°, 14.6°] (height) and [-24.9°, 30.4°] (depth). The combined model for the phantom condition excluded the 8 adjustment responses smaller than 1.

The linear mixed-effects models used for the Bayesian analyses of the height and depth conditions included an intercept and a random effect for participant. We used the same mixed-effects models to carry out a frequentist analysis. For both height and depth conditions, we compared full models (including intercept) with null models that set the intercept to zero.

For the height condition, likelihood ratio tests comparing the full and null models produced p-values less than 0.0005 for both staircase and adjustment data, suggesting that the full model is significantly better than the null model in both cases. For the staircase data alone, the full model yielded a singular fit, so we replaced it with a model without the random effect for participant. For the depth condition, the analogous likelihood ratio tests yielded p-values of 0.01 (staircase) and 0.001 (adjustment).

5 SI Movies

The three SI movies show screen recordings of the scene used for the phantom condition of the VR experiment. The separations between observer and lighthouse are 0 m, 67 and 300 m for the three movies.
